# Acoustic Radiation Force Based Ultrasound Elasticity Imaging for Biomedical Applications

**DOI:** 10.3390/s18072252

**Published:** 2018-07-12

**Authors:** Lulu Wang

**Affiliations:** 1 Department of Biomedical Engineering, School of Instrument Science and Opto-electronics Engineering, Hefei University of Technology, Hefei 230009, China; luluwang2015@hfut.edu.cn; 2 Institute of Biomedical Technologies, Auckland University of Technology, Auckland 1142, New Zealand

**Keywords:** ultrasound elasticity imaging, acoustic radiation force, ultrasonic imaging, tissue stiffness, mechanical properties

## Abstract

Pathological changes in biological tissue are related to the changes in mechanical properties of biological tissue. Conventional medical screening tools such as ultrasound, magnetic resonance imaging or computed tomography have failed to produce the elastic properties of biological tissues directly. Ultrasound elasticity imaging (UEI) has been proposed as a promising imaging tool to map the elastic parameters of soft tissues for the clinical diagnosis of various diseases include prostate, liver, breast, and thyroid gland. Existing UEI-based approaches can be classified into three groups: internal physiologic excitation, external excitation, and acoustic radiation force (ARF) excitation methods. Among these methods, ARF has become one of the most popular techniques for the clinical diagnosis and treatment of disease. This paper provides comprehensive information on the recently developed ARF-based UEI techniques and instruments for biomedical applications. The mechanical properties of soft tissue, ARF and displacement estimation methods, working principle and implementation instruments for each ARF-based UEI method are discussed.

## 1. Introduction

Changes in mechanical properties of tissues also alter pathological state [1]. Previous studies have demonstrated that fibroadenoma tissues and carcinomas are much harder than normal tissues [2]. Stiffness of a material is applied to indicate whether a material is soft or hard. In biology, stiffness has been applied to represent the mechanical properties of a biological substrate [3]. Monitoring tissue stiffness offers helpful information to map internal structures of various biological tissues for medical diagnostic applications [4]. Manual palpation has been widely used as a useful diagnostic method to detect hard tissues surrounded by softer normal tissues since early modern medicine. However, this method is subjective and fails to identify small size lesions and lesions located deep inside the human body. A targeted biopsy is applied to determine tissue elasticity for cancer detection [5]. However, this invasive method is unpleasurable, expensive, complicated, and accuracy remains questionable with sampling errors [6]. Conventional medical imaging techniques such as ultrasound, magnetic resonance imaging (MRI), and computed tomography (CT) cannot directly provide information on the elastic properties of tissues. Fortunately, ultrasound and MRI can measure the internal motion that induced was by external deformations, from which the tissue elasticity can be reconstructed. 

The most non-invasive and cost-effective method, ultrasound imaging, has been widely applied in medical diagnosis since it was first introduced in clinical practice. Ultrasound imaging is one of the most popular screening approaches for evaluating various biological tissues, including breast [7], pancreas [8], prostate [9], kidneys [10], heart [11], liver [12], thyroid [13], and blood vessels of the neck and abdomen [14]. It is a favorite tool used for imaging pregnant women because it can produce detailed images of the fetus and uterus [15]. However, ultrasound imaging has some disadvantages, which include the relatively low contrast between abnormal and surrounding tissues, that it cannot propagate to tissues located deep inside the body at a higher frequency, and it is operator-dependent and subjective. Previous studies have shown that the image quality can be improved by developing new ultrasound imaging approaches [16,17,18,19,20]. 

Developing a non-invasive and cost-effective diagnostic method to monitor tissue stiffness is a subject of interest to many clinicians and scientists [21,22,23,24,25]. Elasticity imaging has been developed to monitor the mechanical properties of soft tissues by using medical imaging modalities, such as ultrasound [26], MRI [27], and optical coherence tomography (OCT) [28]. This last technique involves applying an excitation force over the target tissue to create tissue displacement and thus monitor this tissue displacement to determine the mechanical properties of the tissue, whereas further tissue displacement indicates a softer tissue [29]. The applied excitation force can be induced both internally by ultrasound radiation force [30] and externally by static compression [31] and dynamic vibration [32]. The tissue response can be recorded by ultrasound [31], MRI [33], or acoustic hydrophone [34]. 

In the early 1990s, Ophir et al. first developed a quantitative method, namely elastography, to image the tissue stiffness [31]. This method has been recommended as a clinical diagnostic tool to replace manual palpation for cancerous tissue detection based on the elastic parameters contrast between the normal and abnormal tissues. Over the past two decades, several elastography approaches, such as magnetic resonance elastography (MRE) [33], transient elastography [35], and vibration elastography [36], have been developed and validated for study biological tissues. MRE aims to map the mechanical properties of tissues based on the measured signals in response to an external mechanical vibration, which has been applied to measure living tissues such as the breast [37]. MRE offers high-resolution image and produces more accurate results because it measures all three spatial components of the tissue displacements. However, this method is time-consuming and expensive. Bercoff et al. applied transient elastography to quantitatively map the viscoelastic properties of soft tissues for the clinical diagnosis of breast lesion [38].

Ultrasound elasticity imaging (UEI) has been proposed as a promising imaging tool to map the elastic parameters of soft tissues for the clinical diagnosis of various diseases [39]. During the past two decades, researchers have developed and experimentally tested many new UEI-based techniques for the diagnosis and treatment of diseases. According to the excitation method applied to deform tissue, UEI-based approaches can be classified as internal physiologic excitation, external excitation, and ARF excitation methods [40]. The first UEI is an external excitation method; a transducer can be used to compress the skin in elastography or strain imaging [31]. The tissue can also be deformed by using a physiologic excitation source. Some physiological activities such as arterial pulsation, breathing, and cardiac motion are commonly used as internal sources to induce deformation [41,42,43]. Previous investigations have demonstrated that ultrasound-based internal perturbation techniques could generate a strong force to the tissue located deep inside the body, which can be applied to analyze the mechanical properties of biological tissue [44]. Among these excitation approaches, ARF methods are widely applied to generate shear waves due to the following advantages: a focused beam can be created by an ultrasound system to push tissue where an acoustic window exists; externally created shear waves can encounter the boundary conditions; and the created broadband shear waves by impulsive ARF excitation allow viscoelasticity analysis. ARF-based approaches have smaller strains and less impacted by external boundaries due to the force can be applied directly to the target region. 

This paper reviews the recently developed ARF-based UEI approaches and implementation instruments for biomedical applications. The mechanical properties of biological tissues, ARF and displacement estimation methods, principles, and implementation systems for each ARF-based UEI approach are discussed. The paper is organized as follows. Section 2 presents the mechanical properties of soft tissues; Section 3 describes ARF; Section 4 demonstrates displacement estimation methods; Section 5 shows various ARF-based UEI approaches and implementation instruments; Section 6 presents some current trends and future research directions of ARF-based UEI techniques.

## 2. Mechanical Properties of Biological Tissues 

UEI-based approaches detect abnormal lesions based on the mechanical or elastic properties contrasted between abnormal tissue and healthy tissue. The performance quality is highly related to the force applied to the tissue surface, which is represented as stress ($\sigma =\left\{\sigma_{\mathrm{ij}}\right\}$) [40]:

(1) $$\;f_{i}=\frac{\partial \sigma_{ij}}{\partial x_{j}}=\rho a_{i}\;$$

where f denotes the applied force, a represents the particle acceleration, ρ denotes material density, $\sigma_{ij}$ means Cauchy stress tensor, i, j are spatial vector and tensor subindices

The strain ($\varepsilon =\{\varepsilon_{\mathrm{ij}}$}) is related to the deformed configuration of tissue to its initial reference configuration specifically representing the change in length per unit length, which can be presented by using the placement (u):

(2) $$\;\varepsilon_{ij}=\frac{1}{2}\left(\frac{\partial u_{i}}{\partial x_{j}}+\frac{\partial u_{j}}{\partial x_{i}}+\frac{\partial u_{j}}{\partial x_{i}}\frac{\partial u_{i}}{\partial x_{j}}\right)\;$$

Many assumptions are made to present the tissue behavior under the loading conditions. A linear elastic model can be used to demonstrate the relationship between stress and strain:

(3) $$\;\sigma_{ij}=\lambda \varepsilon \xi_{ij}+2\mu \varepsilon_{ij}\;$$

where λ and μ denote Lamé parameters, $\xi_{\mathrm{ij}}$ denotes a Kronecker delta.

The tissue stiffness is commonly represented by Young’s modulus (*E*) that describes a tissue’s resistance to deformation in uniaxial compression or tension. The shear modulus (*μ*) describes the resistance to shear. The Poisson’s ratio (v) describes the deformation that occurs orthogonal to that of the applied force. The elasticity properties can be defined as:

(4) $$\;E=\frac{\mu \left(3\lambda +2\mu \right)}{\lambda +\mu }\;$$

(5) $$\;v=\frac{\lambda }{2\left(\lambda +\mu \right)}\;$$

(6) $$\;\mu =\frac{E}{2\left(1+v\right)}\;$$

For dense materials, Equation (6) becomes to μ=E/3 when v=0.5.

The following formula can be used to model wave propagation for a linear, elastic, isotropic material:

(7) $$\;\left(\lambda +2\mu \right)\left\{𝛻\left(𝛻\cdot \vec{u}\right)\right\}-\mu \left\{𝛻\times 𝛻\times \vec{u}\right\}=\rho \frac{\partial^{2}\vec{u}}{\partial t^{2}}\;$$

where 𝛻 denotes the spatial gradient operator, 𝛻 and 𝛻× are the divergence and curl operator, respectively. 

The Helmholtz equation can be applied to decompose the displacement as [45]:

(8) $$\;\vec{u}=𝛻\psi +𝛻\times \vec{\theta }\;$$

where ψ is the scalar potential, and $\vec{\theta }$ is the vector potential. 

Separating the components in Equation (8), the longitudinal and transverse propagations can be described as:

(9) $$\;{𝛻}^{2}\psi -\frac{1}{c_{L}^{2}}\frac{\partial^{2}\psi }{\partial t^{2}}=0,\;c_{L}=\sqrt{\frac{\left(\lambda +2\mu \right)}{\rho }}\;$$

(10) $$\;{𝛻}^{2}\vec{\theta }-\frac{1}{c_{T}^{2}}\frac{\partial^{2}\vec{\theta }}{\partial t^{2}}=0,\;c_{T}=\sqrt{\frac{\mu }{\rho }}\;$$

where $c_{L}$ and $c_{T}$ are the longitudinal wave speed and the transverse wave, respectively. These parameters can be represented in terms of elastic moduli.

Various numerical models have been developed to demonstrate the elastic properties of biological tissues. Petrov et al. proposed a parametric identification method to study the elastic parameters of the brain [46]. A Rayleigh damping model has been applied in MRE to simulate soft tissue, which can accurately characterize viscoelastic properties of the brain tissue [47]. Ammari et al. developed a time-reversal imaging model based on Helmholtz decomposition to reconstruct sources in a homogeneous viscoelastic medium [48]. Bretin et al. applied a Voigt model that based on a power law model to describe the viscoelastic properties [49]. 

## 3. Acoustic Radiation Force

ARF has been extensively investigated for numerous biomedical applications, including elasticity imaging [50,51,52,53], molecular screening [54], cancer treatment monitor [55], drug delivery [56], acoustical tweezers [57], and biosensors development [58]. ARF can be classified into two groups: dynamic radiation force and static radiation force. Dynamic radiation force can be induced by ultrasound that has amplitude modulation through time, which has been applied to manipulate various shapes of objects, including spheres [59], and spherical and cylindrical shells [60]. Static radiation force can be created with continuous wave ultrasound to exert a constant force. It is important to note that soft tissue does not support shear stresses at ultrasonic frequencies, and it can be modeled as viscous fluids [61]. Thus, the methods applied by Nyborg [62] and Eckart [63] can be followed to derive an ARF equation. A linear viscous fluid model can be applied to present a dense material (incompressible):

(11) $$\;\sigma_{ij}=-p\delta_{ij}+\left(k-\frac{2}{3}\mu_{f}\right)D\delta_{ij}+\mu_{f}\left(\frac{\partial v_{i}}{\partial x_{j}}+\frac{\partial v_{j}}{\partial x_{i}}\right)\;$$

where $\vec{v}$ denotes the particle speed, p denotes the scalar pressure, D represents aperture width, material constant k denotes the bulk viscosity, and $\mu_{f}$ denotes the shear viscosity. 

For a fluid particle, the acceleration expression can be defined as:

(12) $$\;\vec{a}=\frac{\partial \vec{v}}{\partial t}+\vec{v}\cdot 𝛻\vec{v}\;$$

Combining expressions (1) and (12), the linear viscous fluid model becomes:

(13) $$\;-𝛻p+\left(k+\frac{\mu_{f}}{3}\right)𝛻𝛻\cdot \vec{v}+\mu_{f}{𝛻}^{2}\vec{v}=\rho \left(\frac{\partial \vec{v}}{\partial t}+\vec{v}\cdot 𝛻\vec{v}\right)\;$$

Applying perturbation method to the right side of Equation (13) and keeping up with the second-order terms lead to:

(14) $$\;\rho_{0}\frac{\partial {\vec{v}}_{2}}{\partial t}+\frac{\partial \rho_{1}{\vec{v}}_{1}}{\partial t}+\rho_{0}\left({\vec{v}}_{1}𝛻\cdot {\vec{v}}_{1}+{\vec{v}}_{1}\cdot 𝛻{\vec{v}}_{1}\right)\;$$

where $\rho_{0}$ denotes density, ${\vec{v}}_{1}$ and $\rho_{1}$ present first-order particle speed and first-order particle density, respectively. ${\vec{v}}_{2}$ denotes a higher-order correction term that demonstrates the speed of acoustic streaming.

Taking the time-average of the above equation, then RF is defined as:

(15) $$\;\vec{F}=\rho_{0}\langle {\vec{v}}_{1}𝛻\cdot {\vec{v}}_{1}+{\vec{v}}_{1}\cdot 𝛻{\vec{v}}_{1}\rangle \;$$

Define RF for an exponentially attenuating plane wave as:

(16) $$\;\vec{F}=2\alpha \vec{I}/c_{L}\;$$

where α is the absorption coefficient, $\vec{I}$ presents the time-average intensity, and $c_{L}$ denotes the longitudinal wave velocity.

The left-hand side of Equation (13) would become the following expression if a similar perturbation performed and $𝛻\cdot {\vec{v}}_{2}$ assumed negligible:

(17) $$\;\vec{F}=-𝛻p_{2}+\mu_{f}{𝛻}^{2}{\vec{v}}_{2}\;$$

where $p_{2}$ denotes the second-order pressure. 

The movement is negligible (<1 μm) in conventional ultrasound imaging due to the relatively small ARF. Sufficient ARF can be created using a focused transducer with higher power acoustic pulses. ARF is highly related to the mechanical properties of tissue and characteristics of the transmitted beam, which can be represented by absorption and intensity, and intensity drops with higher attenuation. The depth and beam width of ARF can be described by:

(18) $$\;f_{number}=z/D\;$$

where z and D presents the focal depth and aperture width, respectively. 

## 4. Displacement Estimation Methods

Displacement estimation plays an important step in UEI approaches. The cross-correlation (time domain) and phase-shift approaches are commonly employed to estimate motion displacement. The normalized cross-correlation method offers better performance with reduced bright scatterers, which has been considered as the gold standard displacement estimation method [64]. Kasai et al. developed an auto-correlation method to produce 2D images of color flow in real-time [65]. Loupas et al. proposed a 2D auto-correlation method to improve the system performance; the process accounts for local variations in the center frequency of the received echo for each displacement estimate [66]. Compared with the cross-correlation method, the auto-correlation method offers better performance for monitoring small displacements in ARF-based UEI [67]. Walker et al. developed bias and jitter (σ) estimator to measure tissue displacement accurately [68]. Inaccurate estimate moduli were obtained and noises were produced in the images when Jitter has been selected in a range of 1~5 μm [69]. Doherty et al. applied the ARF-based harmonic method for feature detection with improved jitter reduction [70].

## 5. ARF-Based UEI Techniques 

According to the excitation source methods applied to the tissue, UEI-based approaches can be classified into three groups: internal excitation, external excitation, and ARF excitation. Based on the duration of excitation, ARF excitation methods can be further classified into three groups: quasi-static, harmonic elasticity imaging (HEI), and transient elasticity imaging (TEI). Acoustic streaming in diagnostic ultrasound (ASDU) and sonorheometry are the two main types of quasi-static methods. Various HEI approaches and implementation systems, such as vibro-acoustography (VA), harmonic motion imaging (HMI), shear wave ultrasound dispersion ultrasound vibrometry (SDUV), and crawling wave spectroscopy (CWS), have been developed for biomedical imaging applications.

### 5.1. Quasi-Static Methods

The quasi-static method was the first elastography technique developed based on a quasistatic deformation of the tissue. Compression applied to the tissue, and the induced strain image is extracted from the difference between the reference image and the compressed image. The displacement is often computed by using B-mode ultrasound images (correlation method). Strains are computed by spatial derivation following one or two directions for the most evolved approaches. 

In 1991, elastography was first developed by Ophir et al. to map the elasticity of biological tissues [31]. In this study, a transducer was coupled to the target tissue to record echo signal for a given time, and then the transducer was pressed into the tissue to record another echo signal. The tissue displacements were estimated from these two echo signals, and the strain image was produced using the relation between strain and tissue displacement. This method was conceptualized by using springs; the stiffest spring will compress the least. In 1995, Plewes et al. investigated MRE that applies a phase-contrast approach [71]. The quasi-static methods by MRI require much longer acquisition time than quasi-static methods by ultrasound (up to 15 times longer), but MRE images show better contrast.

Nightingale et al. applied ASDU to separate fluid-filled lesions and solid lesions in breast tissue [72]. Viola et al. used sonorheometry to measure the properties of coagulation by monitoring the blood fluid response to a quasi-static ARF excitation [73]. The measurements can be fitted to a viscoelastic Voigt fluid model to obtain the time-constant, a force-free parameter that provides a quantitative estimate of the rate of stiffening. Recently, an adaptive force sonorheometry has been developed for the assessment of tissue stiffness [74]. This method adjusts the pulse repetition frequency of the applied excitations based on the maximum displacement. Quasi-static methods offer a relatively good estimation of elastic parameters, which have been applied to detect breast lesions [75]. The main drawbacks of quasi-static methods include specific quantification and that they are operator dependent. In addition, quasi-static methods produce artifacts and are not applicable to tissues located deep inside the body. 

### 5.2. Harmonic Elasticity Imaging Methods

**Figure 1:**
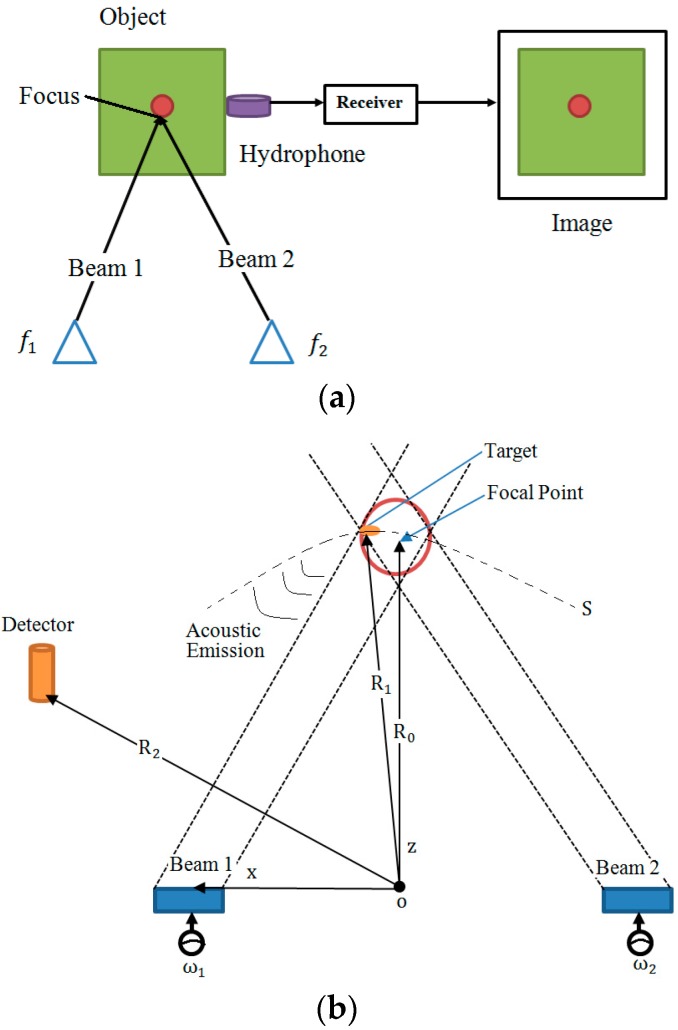
(**a**) Diagram of a Vibro-Acoustography (VA) imaging system; (**b**) The plane wave model for VA beamforming.

Ultrasound-Stimulated Vibro-Acoustography (USVA) was developed by Fatemi and Greenleaf to map the acoustic response of an object [34]. The method was experimentally tested on excised human iliac arteries, and the results were compared with X-ray imaging. The potential applications of USVA include non-destructive evaluating of materials and medical diagnostic imaging. As shown in Figure 1, a simplified USVA system consists of two confocal ultrasound transducers to generate signals to the target object at different frequencies, and a sensitive hydrophone to measure the generated acoustic emission from the object [76]. This technique has been applied to measure human breasts in vivo and results have shown that it can detect microcalcifications regardless of the breast density [77]. Small calcifications (<1 mm) were successfully identified by using VA. Compared with the single frequency VA, the multi-frequency VA offers more useful information on the target object without increasing operation time [78]. 

To date, VA has been applied for imaging various tissues including breast [77], arterial vessels [79], prostate [80], liver [81], bone [82], and heart [83]. About 78.4% of micro-calcifications in excised tissues could be successfully detected using VA [76]. Compared with ultrasound and mammography, VA produces more accurate results for breast lesion detection. The method presented in [84] combined VA and mammography to increase the impact of both methods. Pislaru et al. applied VA to measure pig arteries [85], and results showed that the sensitivity and specificity of VA for detecting calcifications are 100% and 86%, respectively. VA has some advantages and disadvantages. It is speckle-free, high contrast and high sensitivity due to high sensitivity hydrophones are involved in the measurement system. However, the match solution medium is required for experimental validation of VA technique which also increases the difficulty and operating cost. The image quality can be reduced when the absorption and attenuation of the organs increased. Alternatively, the image quality can be improved by using the X-focal system with two transducers, an annular array of multiple rings, and combining data collected in different ways. 

Konofagou et al. proposed HMI for imaging localized harmonic motion by using two focused ultrasound transducers [86]. An HMI system usually consists of a single focused ultrasound transducer and an ultrasound imaging transducer. An amplitude-modulated waveform is applied to produce an internal vibration that is used to interrogate the mechanical properties of tissue. Finite-element model (FEM) and Monte-Carlo model have been applied to simulate the oscillatory displacement within an applied force frequency range of 200–800 Hz. Maleke et al. investigated the amplitude-modulated HMI for the early diagnosis of tumors [87]. The force was applied at the specific nodes within the focal region using a FEM. However, the method did not use realistic RF because the 2D acoustic pressure was not considered. Heikkilä et al. developed a localized HMI (LHMI) model to numerical analyze the LHMI for detecting a lesion, and the authors also demonstrated the theory in vivo [88]. LHMI applies a series of quasi-static excitations at a specific rate. Two configurations, a 1D linear array transducer and two single-element transducers, were investigated for sonication and imaging. HMI has been applied for monitoring radiofrequency ablation in liver [89] and breast [90], as well as imaging biological tissues [91].

More recently, Top et al. proposed a new 2D imaging method namely harmonic motion microwave Doppler imaging (HMMDI) that combines microwave and ultrasound imaging approaches to identify lesions [92]. Like the HMI approach, the HMMDI system consists of a focused ultrasound probe to induce local vibrations inside the tissue, a microwave transmitter to transmit microwave signals to the tissue, and a microwave receiver to measure the backscattered fields from the tissue. The measured signals contained amplitude and phase information that was highly related to dielectric properties, displacement, and volume of the vibrating region. The backscattered energy level changed when the vibrating region contained a region that has a different mechanical and or electrical properties than the background. The authors have numerically and experimentally tested the method on various breast models and phantoms. The results demonstrated that HMMDI has a potential to detect breast lesions inside fibro-glandular breast tissue. This method provided more information on the target object than microwave or elasticity imaging alone due to the measurements are dependent on both mechanical and electrical parameters of the object. However, clinical validations on human subjects are required in the future. The authors further numerically investigated a 3D HMMDI model by including the axial plane cuts to improve the image resolution. In their study, the measured HMMDI data from breast lesion inside homogeneous tissue was analyzed as a function of lesion location on three orthogonal planes using FDTD method. This method could produce millimeter-resolution image.

Chen et al. proposed SDUV that combines VA and SWEI to generate monochromatic shear waves to monitor shear wave speed dispersion in a viscoelastic medium [93]. They used VA to create harmonic shear waves and utilized SWEI to measure off-axis waves using pulse-echo ultrasound technique. The same research team also developed a pulse sequence method to analysis the push and detection function using one array transducer. The proposed pulse sequence method was validated on swine liver. The measured wave speed differences at different frequencies were analyzed to assess the dispersion. SDUV has been applied in vitro to measure the shear wave velocity in the kidney [94] and prostate [95], and in vivo to study dispersion in liver [96]. The limitations of SDUV include the method measures single point that causes defects in producing images. SDUV measurement system may not provide enough energy intensity to the tissue located in deep of body to generate sufficient vibration. The new ultrasonic transducers are expected to measure lesions at depths of 5~7 cm from the body. Additionally, tiny tumors cannot be detected using SDUV, as the technique assumes a homogeneity for the measurement site. 

Hah et al. numerically and experimentally demonstrated that crawling waves could be created from focused beams that create RF excitation in the tissue [97]. The slow-moving crawling wave is created between two sources of slightly offset frequencies that are measured by using the Doppler method. The tissue stiffness can be quantitatively estimated from the crawling wave velocity that is related to the mechanical properties. CWS has been applied to develop commercial ultrasound imaging systems such as GE Logiq 9 and to detect cancerous tissue in excised prostate glands [98]. 

### 5.3. Transient Elasticity Imaging Methods

The tissue elasticity can be determined by measuring the transient tissue deformation applied by an impulsive ARF excitation. The tissue displacement can be spatially and temporally monitored using pulse-echo ultrasound technique. This section reviews the recently developed TEI approaches for monitoring tissue deformation, including acoustic radiation force impulse (ARFI) imaging, shear wave elasticity imaging (SWEI), supersonic shear imaging (SSI), shear wave spectroscopy (SWS), and spatially modulated ultrasound radiation force (SMURF). 

Nightingale et al. proposed ARFI imaging to investigate liver fibrosis [51]. In this study, the authors applied a short-duration ARF to create localized tissue displacements and used a cross-correlation method to track the tissue displacements during relaxation. The ARFI imaging system uses the same ultrasound transducer to induce and measure deformation response. A typical ARFI contains three types of pulses, including reference pulses, pushing pulses, and tracking pulses. Reference pulses establish a baseline position of the tissue before the ARF excitation, pushing pulses to create the ARF to induce localized deformation; and tracking pulses monitor the deformation response and recovery of the soft tissue. The ensemble of these three types of pulses can be translated across the aperture to acquire 2D information. The displacements from single reference pulse and all tracking pulses can be computed using cross-correlation and phase-shift methods to achieve time-domain displacements that can be applied to determine tissue stiffness. Images of tissue displacements can be produced at a specific time such as maximum tissue displacement. In general, softer tissues will displace farther, take longer to reach a maximum displacement, and recover more slowly than stiffer tissues. ARFI has been applied to identify breast lesions [99] and lymph nodes [100].

Sarvazyan et al. applied SWEI to monitor the stiffness of tissue [51]. They employed a high-intensity focused ultrasound piston to generate shear waves of soft tissues. In an ultrasound-based SWEI system, shear waves can be created by using an external mechanical vibration or ARF from a focused ultrasound beam. Nightingale et al. experimentally evaluated SWEI for displacement estimation by using a single ultrasound transducer to generate impulsive ARF excitation in tissue and utilized pulse-echo ultrasound to track the movements during relaxation [52]. The proposed method analyzed the mechanical properties of organs, which provided a promise for disease detection. SWEI has been applied for imaging various tissues include liver, cardiac tissue, and prostate.

**Figure 2:**
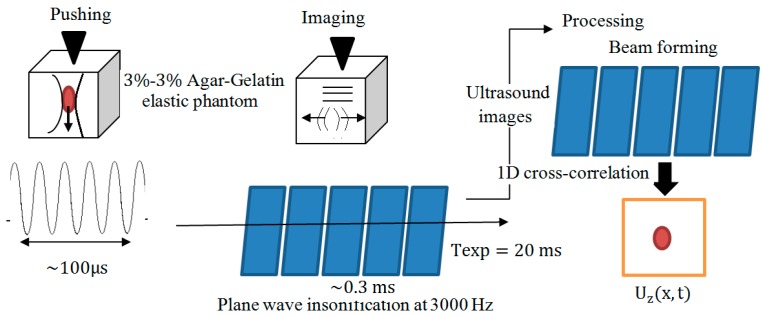
Schematic drawing of supersonic shear imaging (SSI).

ARF-based methods usually induce small displacements. To solve this problem, Bercoff et al. proposed SSI to quantitatively analyze the elasticity and viscosity of materials [53]. This method uses multiple moving ARFIs to create two plane shear waves at supersonic speed and uses an ultra-fast ultrasound scanner to measure the displacements (see Figure 2). SSI has been experimentally investigated to study liver fibrosis [101], breast [4], and muscle [102]. Tanter et al. reported initial clinical trials results of using SSI for imaging of breast lesion [4]. In their study, a modified 1D array transducer was applied to produce a radiation force that resulted in transient shear wave propagation. An ultra-fast imaging sequence was performed to obtain successive radiofrequency signals at high frame rates. SSI successfully detected breast masses and offered quantitative information on the stiffness of masses. Previous studies showed that SSI offers more accuracy for detecting liver cirrhosis than fibrosis, while ARFI offers similar accuracy for detecting liver cirrhosis and fibrosis. SSI can produce an image in less than a half minute. However, the technique requires ultra-fast ultrasound scanner to produce high-resolution images which slow down the development of commercial SSI instruments due to the limited speed of ultrasound scanners. 

SWS has been applied for in vivo evaluation of human soft tissues [103]. SWS generates a quasi-plane shear wave to induce displacements that can be measured across the region of interest (ROI) at ultrafast rates. SWS aims to estimate the shear wave speed for individual frequency components, which is very different from SWEI and SSI approaches that attempt to access the shear wave group velocity. However, this method produces poor SNR due to the energy within the individual signal is smaller than that of the whole message. The SNR can be improved by assuming homogeneity within a significant ROI. 

McAleavey et al. proposed SMURF to monitor the shear modulus of tissue by measuring shear waves propagated at the temporal frequency [104]. An ultrasound scanner and VF7-3 linear array were used to scan and record the shear modulus of gelatine phantom and liver tissue at 4.21 MHz. SMURF identified small size lesion (2.5 mm) embedded in the organ phantom and inclusion embedded in the liver. Different from SDUV and SSI, SMURF tracking takes place at a single location. The location of a speckle at depth may bias the arrival time estimate of a shear wave from one pushing location. SMURF method could rapidly estimate shear modulus of tissue.

## 6. Discussion and Future Work

Numerous UEI approaches have been extensively investigated for biomedical applications over the past two decades. ARF-based UEI techniques do not dependent on operators, thus facilitating operator-independent data acquisition. Each ARF-based UEI method offers a unique mechanism of image contrast that is helpful for identifying and monitoring disease. SWEI, SSI, SWS, SMURF, SDUV, and CWS are qualitative methods, which are more suitable for structural imaging and analysis of tissues. While quantitative methods are more helpful to detect abnormal tissues that are not confined to a target region. Quasi-static and harmonic methods are typically assumed local homogeneity, which simplifies the simulation validation. However, this assumption cannot produce accurate estimations near boundaries. The ARF-based methods can determine the tissue elasticity without obtaining the values in the boundaries. Since biological tissues are not entirely homogeneous, there have been efforts to avoid assuming local homogeneity. However, such methods require a high computational cost.

HMI and VA are confined to smaller regions due to long data acquisition duration and more suitable for detecting suspected sites where a biopsy may be performed for closer examination. The potential applications of VA include biomedical imaging and material evaluation fields. In medical imaging, investigators studied VA for imaging of breast phantoms and human breasts, human vessels, in vivo imaging of animal arteries, image brachytherapy seeds positioned in the excised prostate tissue, liver tissue, bone, and detection of calcium deposits on heart valves. Compared with pulse-echo imaging methods, VA enhanced the detectability of brachytherapy seeds. Mitri et al. compared the integrated optical density with pulse-echo and VA imaging [80]. Results indicated that the integrated optical density of the pulse-echo drops when removing transducers from the incident concerning the seeds. Recently, VA has been applied to identify small tears [105]. Material evaluation is also another exciting application field of VA, including the mechanical parameter, imaging, and non-destructive validations of materials. The future investigations of VA include the following areas.

- Analysis methods and modeling approaches should be refined to solve the spatial distribution of viscoelastic properties.
- Develop a 3D VA method and instrument to produce 3D images. Most of existing VA techniques produce 2D images, which cannot produce accurate image results of a 3D object. Recently, Kamimura et al. developed a method based on VA and B-mode techniques to produce 3D images of bone [106]. However, clinical validations of the proposed method are required in the future.
- Optimization of electronic beam forming with array transducers to improve image resolution and sensitivity, as well as reduce the scanning time is another exciting study area. Mayo Clinic developed linear and phased arrays to study VA clinically. Design and validation of random and spiral linear and nonlinear arrays should be helpful for this work.
- Method validations on human subjects are required to prove the possibility and safety of VA for imaging living tissues, such as heart, breast, prostate, and thyroid.
- Develop a commercial VA system includes a high-sensitive transducer is the natural pathway of this work.

The newly developed HMMDI approach combines the benefits of microwave imaging and HMI techniques, which offers the potential to become a powerful medical imaging tool for breast cancer detection [92]. However, this method requires further clinical trial validations and the development of commercial instruments for use in clinical trials in the future.
